# Expanding the Minimally Invasive Approach towards the Ascending Aorta—A Practical Overview of the Currently Available Techniques

**DOI:** 10.3390/medicina59091618

**Published:** 2023-09-07

**Authors:** Florian Helms, Bastian Schmack, Alexander Weymann, Jasmin Sarah Hanke, Ruslan Natanov, Andreas Martens, Arjang Ruhparwar, Aron-Frederik Popov

**Affiliations:** Division for Cardiothoracic, Transplantation and Vascular Surgery, Hannover Medical School, 30625 Hannover, Germany

**Keywords:** minimally invasive surgery, aortic surgery, ascending aorta

## Abstract

Minimally invasive techniques have gained immense importance in cardiovascular surgery. While minimal access strategies for coronary and mitral valve surgery are already widely accepted and often used as standard approaches, the application of minimally invasive techniques is currently expanded towards more complex operations of the ascending aorta as well. In this new and developing field, various techniques have been established and reported ranging from upper hemisternotomy approaches, which allow even extensive operations of the ascending aorta to be performed through a minimally invasive access to sternal sparing thoracotomy strategies, which completely avoid sternal trauma during ascending aorta replacements. All of these techniques place high demands on patient selection, preoperative planning, and practical surgical implementation. Application of these strategies is currently limited to high-volume centers and highly experienced surgeons. This narrative review gives an overview of the currently available techniques with a special focus on the practical execution as well as the advantages and disadvantages of the currently available techniques. The first results demonstrate the practicability and safety of minimally invasive techniques for replacement of the ascending aorta in a well-selected patient population. With success and complication rates comparable to classic full sternotomy, the proof of concept for minimally invasive replacement of the ascending aorta is now achieved.

## 1. Introduction

Over the last two decades, minimally invasive approaches have gained immense importance in cardiothoracic surgery [1]. Over the last decade, the minimally invasive access has more and more become the standard approach for coronary revascularization and mitral valve surgery as well as for aortic valve and aortic root replacement [2,3,4,5]. Here, Shrestha et al. reported comparable peri- and postoperative outcomes in 42 patients undergoing David procedure through an upper hemisternotomy compared to 178 patients operated through full sternotomy, while operation and cross clamp times were slightly longer [6]. As the next logical step, minimally invasive approaches for more complex operations including the supracommissural ascending aorta have currently moved into the center of attention. However, highly variable morphology and position of the ascending aorta and a wide variety of different aortic pathologies require elaborate planning and highly sophisticated strategies if using a minimally invasive approach. This narrative review gives a practical overview of the currently available strategies for patient selection, preoperative planning, access, and cannulation as well as myocardial and cerebral protection in minimally invasive surgery of the ascending aorta. The impact of less invasive approaches to ascending aortic surgery on operation times, as well as complications and outcomes compared to the classic full sternotomy strategy, are also briefly summarized.

## 2. Patient Selection

As a newly developed technique, minimally invasive strategies for operations of the ascending aorta were initially applied in a well selected patient population. As an example, Starmolynski et al. reported a less invasive approach to ascending aorta and aortic root surgery in 167 patients with a low or moderate risk profile and a mean EuroScore II of 2.58 [7]. Likewise, Byrne et al. excluded elderly and high risk patients from minimally invasive approaches to the ascending aorta with the intention of minimizing bypass and cross clamp times in these cases [8]. While some groups report successful application of a minimally invasive access not only for elective but also for urgent cases [9,10], broad consensus exists among all reporting authors that emergency operations of the ascending aorta should still be addressed by full sternotomy to facilitate extension of the operation if necessary. Since access is usually limited to the upper mediastinum in minimally invasive approaches targeting the ascending aorta, the need for concomitant coronary bypass grafting or mitral valve surgery must be considered a contraindication for minimally invasive access in aortic surgery [8,11]. On the other hand, concomitant aortic valve replacement is possible and frequently performed using the minimally invasive approach [8,12]. Likewise, previous cardiac operations do not constitute a contraindication for minimally invasive approaches to the ascending aorta [8,10,13,14]. Svensson et al. report an experience of 54 consecutive patients including 33% redo-cases with short-term results that are comparable to re-operations via full sternotomy [14]. They consider the minimally invasive approach to be particularly suitable for re-operations, as it may be possible to completely avoid preparation of the right and anterior sides of the heart, which simplifies reoperations especially after previous coronary- or AV-valve surgery.

In addition to patient selection, it is furthermore noteworthy that all reports of successful application of the minimally invasive approach to the ascending aorta were published from high-volume centers with specialized aortic and minimally invasive surgery programs. Involved surgeons had substantial experience in minimally invasive strategies for aortic valve surgery and root replacement before extending this access strategy towards more complex operations of the ascending aorta.

## 3. Preoperative Diagnostics and Planning

Preoperative contrast enhanced computed tomography (CT) of the thoracic aorta is the central diagnostic tool for preoperative assessment of patients that are potentially suitable for minimally invasive ascending aortic surgery. Since the positioning of the aortic cross clamp is limited to a certain area of the distal ascending aorta and tactile assessment of the aortic wall for potential atherosclerotic plaques is difficult through the minimally invasive access, CT-radiographic assessment of aortic sclerosis and potential clamp areas is essential [15]. Additionally, Starmolynski et al. advocate for the use of CT-angiography to determine the retrosternal aortic positions, since abnormal ascending aortic positions might require a more extensive hemisternotomy or even full sternotomy [7]. They report a right-sided ascending aorta in 95%, central position in 4% and left-sided ascending aorta position in 1% of their cases. Moreover, preoperative echocardiography and coronary angiography is usually recommended to assess the need for concomitant procedures that might prohibit the use of a minimally invasive approach [7,15,16].

## 4. Operative Techniques

### 4.1. Access Sides

**Figure 1:**
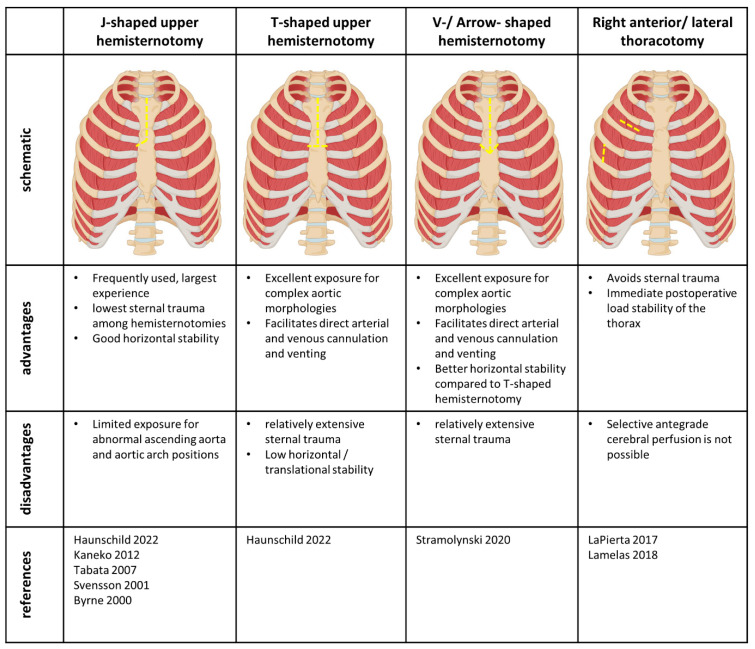
Schematic representation and summary of the currently available access techniques. Yellow dotted lines represent the respective incisions [6,7,9,10,11,13,14,17].

Various access sides have been described ranging from the classical J-shaped upper hemisternotomy to a completely sternal sparing approach using right-sided thoracotomies. Figure 1 gives an overview of the currently available access strategies as well as their potential benefits and disadvantages. The most common minimally invasive access to the ascending aorta is the right-sided J-shaped upper hemisternotomy with division of the apical sternum to the third or fourth intercostal space [8,9,10,12,14]. With the left side of the sternum intact, sternal trauma is limited and horizontal translational stability of the sternum is provided. However, access to more complex aortic morphologies or abnormal right-sided position of the ascending aorta might be limited through this incision [7]. To enhance exposure, the horizontal incision can be carried out through both sides of the sternum resulting in the so-called T-shaped upper hemisternotomy [12]. While this technique may facilitate addressing a wider range of complex aortic pathologies and facilitates direct aortic and right atrial cannulation as well as venting through the right upper pulmonary vein, the continuous horizontal division of the sternum may cause horizontal translational instability. Using both T- and J- shaped hemisternotomies for combined supracommissural ascending aorta replacement and aortic valve replacement, Haunschild et al. report sternal instabilities in 2.6% of patients after minimally invasive surgery [12]. Considering this, the so-called V-shaped or arrow-shaped upper hemisternotomy might be advantageous. This too facilitates excellent exposure for complex aortic morphologies and abnormally positioned ascending aortas. In contrast to the T-shaped incision, the horizontal division of the sternal halves is performed in an angled fashion, which provides translational stability in both, horizontal and vertical directions [7]. Again, the third or fourth intercostal spaces are used for the horizontal incision. Since both sternal halves are divided, the T- or V-shaped hemisternotomies cause a more extensive sternal trauma compared to the standard J- shaped incision.

In a more recent development, sternotomy is completely avoided by using a right anterior thoracotomy. For this technique, a 6 cm incision is made in the second or third intercostal space in the mid-clavicular line [11,15]. Consequently, the third (and fourth) chondrocostal cartilages are dislocated to gain horizontal access to the aorta. In addition to that, Lamelas et al. reported a variation of this approach using a right lateral thoracotomy in the anterior axillary line in the fourth intercostal space [11]. These sternal- sparing techniques have the great advantage of avoiding compromising the load stability of the thorax. This potentially facilitates quicker mobilization and physical recovery after surgery. Moreover, cosmetic results of this approach are excellent. On the other hand, exposure of the supra-aortic vessels is strongly limited through the thoracotomy approach, which makes selective anterior cerebral perfusion impossible. However, Lamelas et al. report successful use of retrograde cerebral perfusion with the right thoracotomy access. To address potential postoperative pain resulting from spreading or dislocation of the ribs in thoracotomy approaches, direct intraoperative local anesthetic infiltration or nerve blocks may be used [11].

### 4.2. Cannulation

Various techniques have been described for cardiopulmonary bypass initiation in minimally invasive ascending aortic repair. Most often, direct aortic cannulation via the primary incision is used for arterial cannulation [7,8,9,10,12]. In case of re-operations or when using the right thoracotomy access, axillary artery cannulation [8,9,10,11] or femoral artery cannulation [8,9,11,12,15] may be performed as well. However, most groups prefer antegrade arterial perfusion via direct aortic cannulation or axillary artery cannulation over femoral artery perfusion due to the potential risk of retrograde thrombus dislocation in the latter. Svensson et al., who reported the largest series of re- operations through the minimally invasive access, use right subclavian artery cannulation or femoral artery cannulation for redo cases [14].

Venous cannulation can be performed directly though the right atrium using the hemisternotomy approaches. Typically, the venous cannula is guided through a separate subxiphoidal incision, which is later used for a drainage tube [7,14]. Alternatively, venous drainage can be performed by femoral vein cannulation and echocardiography-guided positioning of a two-stage venous cannula [10,11,15]. In complex redo cases, Byrne et al. propose cannulation of the left innonimate vein and advancing the venous cannula into the right atrium using the over-the-wire technique to avoid preparation of the right side of the heart [8].

### 4.3. Venting and Cross-Clamping

Even in minimally invasive operations, left ventricular venting can often be achieved in the standard fashion via the right superior pulmonary vein through the main incision [7,12,15]. Alternatively, some authors prefer pulmonary artery venting [9,10] or perform direct left ventricular transvalvular venting through the aortic valve after aortotomy [8,9,10]. The aortic cross clamp is usually applied through the main incision [7], while other cross-clamping techniques developed for minimally invasive aortic valve replacement or mitral valve surgery such as the “Glauber clamp” [17] or insertion of a Chitwood^®^-clamp via an additional access side [18] may also be applicable in ascending aortic surgery.

### 4.4. Myocardial and Cerebral Protection

Cardioplegia can be administered in a standard fashion via the ascending aorta [8], as direct antegrade cardioplegia though the coronary ostia [7,15] or in a retrograde fashion through the coronary sinus [14]. In the case of re-operations, Byrne et al. use a transjugular coronary sinus catheter for retroinfusion [8]. In terms of temperature management, all authors propagate comparably aggressive cooling strategies. Perrotta et al. recommend additional topical cooling by slush ice for myocardial protection [19]. Others used systemic cooling to lower than 28 °C for myocardial protection [8], which can be even lowered to 20 °C if circulatory arrest is necessary [15]. However, as the minimally invasive techniques become more standardized in the future, mild hypothermia may be just as suitable for less invasive strategies as they are established in classical open cases [20]. Additionally, selective antegrade cerebral perfusion can be performed using the hemisternotomy approaches [13]. In contrast to that, when using the sternal sparing approach only retrograde cerebral perfusion is practicable [11,15].

## 5. Outcome

Due to the heterogeneity of concomitant procedures performed in addition to minimally invasive replacement of the ascending aorta in current reports, direct comparisons of the outcomes of different minimally invasive approaches are not practicable with the available data to date. However, some authors used propensity score matching to compare their strategy using the minimally invasive approach to the standard full sternotomy. Here, Haunschild et al. found no significant differences between partial and full sternotomy for procedure- and cross-clamp time, long-term survival or reoperation rates after 2:1 propensity score matching of 117 patients undergoing minimally invasive supracommissural or standard ascending aortic replacement in combination with aortic valve replacement [12]. In another matched analysis, Tabata et al. reported a reduction in the length of hospital stays by one day and less blood product requirement when using an upper J-shaped hemisternotomy compared to full sternotomy. Lamelas et al. reported equal cross-clamp and bypass times using the sternal sparing thoracotomy approach compared to full sternotomy, while the mean circulatory arrest time was longer. They too report a reduction in postoperative hospital stays by one day and lower rates of red blood cell transfusions [11]. In contrast, although rare overall, re-thoracotomy for bleeding was occasionally necessary in almost all series [8,9,19].

## 6. Limitations

The small number of available studies on this newly developed technique carries the risk of publication bias. In particular, reporting of postoperative pain management, incidence of pneumothorax, or occult bleeding is often insufficient. Moreover, distinct heterogeneity with respect to concomitant aortic valve or proximal aortic arch replacement in addition to the ascending aortic operation can be observed among the reports published to date. While there is a broad evidence basis for minimally invasive aortic root and aortic valve replacements, currently available evidence focusing on the supracommissural aorta is either published as experience reports [7,8,10,14,15] or as retrospective propensity matched analyses, in part with a historical control group [9,11,12], while no prospective analysis was performed to date.

## 7. Conclusions and Future Perspective

All the techniques detailed in this review have individually and collectively achieved the proof of concept for minimally invasive approaches to the ascending aorta. In the hands of highly experienced surgeons, ascending aortic replacement via a minimally invasive access is save and does not increase procedure or bypass times. Before broad clinical application and guideline recommendation can be targeted, this new technique has to be proven save and sufficient in prospective trials as well.

## Data Availability

Not applicable.

## References

[1] Iribarne A., Easterwood R.M., Chan E.Y., Yang J., Soni L., Russo M.J., Smith C.R., Argenziano M., Bostock I.C., Nammalwar S. (2011). The golden age of minimally invasive cardiothoracic surgery: Current and future perspectives. Future Cardiol..

[2] Monsefi N., Risteski P., Miskovic A., Moritz A., Zierer A. (2018). Midterm Results of a Minimally Invasive Approach in David Procedure. Thorac. Cardiovasc. Surg..

[3] Jahangiri M., Hussain A., Akowuah E. (2019). Minimally invasive surgical aortic valve replacement. Heart.

[4] Abu-Omar Y., Fazmin I.T., Ali J.M., Pelletier M.P. (2021). Minimally invasive mitral valve surgery. J. Thorac. Dis..

[5] Sun L., Zheng J., Chang Q., Tang Y., Feng J., Sun X., Zhu X. (2000). Aortic root replacement by ministernotomy: Technique and potential benefit. Ann. Thorac. Surg..

[6] Shrestha M., Kaufeld T., Shrestha P., Martens A., Rustum S., Rudolph L., Krüger H., Arar M., Haverich A., Beckmann E. (2022). Valve-sparing David procedure via minimally invasive access does not compromise outcome. Front. Cardiovasc. Med..

[7] Staromłyński J., Kowalewski M., Sarnowski W., Smoczyński R., Witkowska A., Bartczak M., Drobiński D., Wierzba W., Suwalski P. (2020). Midterm results of less invasive approach to ascending aorta and aortic root surgery. J. Thorac. Dis..

[8] Byrne J.G., Karavas A.N., Cohn L.H., Adams D.H. (2000). Minimal access aortic root, valve, and complex ascending aortic surgery. Curr. Cardiol. Rep..

[9] Tabata M., Khalpey Z., Aranki S.F., Couper G.S., Cohn L.H., Shekar P.S. (2007). Minimal access surgery of ascending and proximal arch of the aorta: A 9-year experience. Ann. Thorac. Surg..

[10] Kaneko T., Couper G.S., Borstlap W.A., Nauta F.J., Wollersheim L., McGurk S., Cohn L.H. (2012). Minimal-access aortic valve replacement with concomitant aortic procedure: A 9-year experience. Innovations.

[11] Lamelas J., Chen P.C., Loor G., LaPietra A. (2018). Successful Use of Sternal-Sparing Minimally Invasive Surgery for Proximal Ascending Aortic Pathology. Ann. Thorac. Surg..

[12] Haunschild J., van Kampen A., von Aspern K., Misfeld M., Davierwala P., Saeed D., Borger M.A., Etz C.D. (2022). Supracommissural replacement of the ascending aorta and the aortic valve via partial versus full sternotomy-a propensity-matched comparison in a high-volume centre. Eur. J. Cardiothorac. Surg..

[13] Svensson L.G. (2002). Progress in ascending and aortic arch surgery: Minimally invasive surgery, blood conservation, and neurological deficit prevention. Ann. Thorac. Surg..

[14] Svensson L.G., Nadolny E.M., Kimmel W.A. (2001). Minimal access aortic surgery including re-operations. Eur. J. Cardiothorac. Surg..

[15] LaPietra A., Santana O., Pineda A.M., Mihos C.G., Lamelas J. (2014). Outcomes of aortic valve and concomitant ascending aorta replacement performed via a minimally invasive right thoracotomy approach. Innovations.

[16] Deschka H., Erler S., Machner M., El-Ayoubi L., Alken A., Wimmer-Greinecker G. (2013). Surgery of the ascending aorta, root remodelling and aortic arch surgery with circulatory arrest through partial upper sternotomy: Results of 50 consecutive cases. Eur. J. Cardiothorac. Surg..

[17] Celmeta B., Viva T., Bisogno A., Bruno V.D., Miceli A., Glauber M. (2023). Detachable Aortic Clamp for Minimally Invasive Cardiac Surgery. Surg. Technol. Int..

[18] Sansone F., Ceresa F., Patanè F. (2013). Transcutaneous insertion of the Chitwood^®^ clamp in case of minimally invasive cardiac surgery. Personal experience. Il G. Chir.-J. Ital. Surg. Assoc..

[19] Perrotta S., Lentini S. (2009). Ministernotomy approach for surgery of the aortic root and ascending aorta. Interact. Cardiovasc. Thorac. Surg..

[20] Zierer A., El-Sayed Ahmad A., Papadopoulos N., Moritz A., Diegeler A., Urbanski P.P. (2012). Selective antegrade cerebral perfusion and mild (28 °C–30 °C) systemic hypothermic circulatory arrest for aortic arch replacement: Results from 1002 patients. J. Thorac. Cardiovasc. Surg..

